# Plasma-Generated Nitric Oxide Water Mediates Environmentally Transmitted Pathogenic Bacterial Inactivation via Intracellular Nitrosative Stress

**DOI:** 10.3390/ijms24031901

**Published:** 2023-01-18

**Authors:** Shweta B. Borkar, Manorma Negi, Neha Kaushik, Shaik Abdul Munnaf, Linh Nhat Nguyen, Eun Ha Choi, Nagendra Kumar Kaushik

**Affiliations:** 1 Plasma Bioscience Research Center, Department of Electrical and Biological Physics, Kwangwoon University, Seoul 01897, Republic of Korea; 2 Department of Biotechnology, College of Engineering, The University of Suwon, Hwaseong 18323, Republic of Korea; 3 Laboratory of Plasma Technology, Institute of Materials Science, Vietnam Academy of Science and Technology, 18 Hoang Quoc Viet, Hanoi 100000, Vietnam

**Keywords:** microwave plasma, antibacterial activity, intracellular nitrosative stress, membrane damage, reactive oxygen, nitrogen species

## Abstract

Over time, the proportion of resistant bacteria will increase. This is a major concern. Therefore, effective and biocompatible therapeutic strategies against these bacteria are urgently needed. Non-thermal plasma has been exhaustively characterized for its antibacterial activity. This study aims to investigate the inactivation efficiency and mechanisms of plasma-generated nitric oxide water (PG-NOW) on pathogenic water, air, soil, and foodborne Gram-negative and Gram-positive bacteria. Using a colony-forming unit assay, we found that PG-NOW treatment effectively inhibited the growth of bacteria. Moreover, the intracellular nitric oxide (NO) accumulation was evaluated by 4-amino-5-methylamino-2′,7′-dichlorofluorescein diacetate (DAF-FM DA) staining. The reduction of viable cells unambiguously indicates the anti-microbial effect of PG-NOW. The *soxR* and *soxS* genes are associated with nitrosative stress, and *oxyR* regulation corresponds to oxidative stress in bacterial cells. To support the nitrosative effect mediated by PG-NOW, we have further assessed the *soxRS* and *oxyR* gene expressions after treatment. Accordingly, *soxRS* expression was enhanced, whereas the *oxyR* expression was decreased following PG-NOW treatment. The disruption of cell morphology was observed using scanning electron microscopy (SEM) analysis. In conclusion, our findings furnish evidence of an initiation point for the further progress and development of PG-NOW-based antibacterial treatments.

## 1. Introduction

The emergence of new resistance mechanisms and the spread of antibiotic-resistant bacteria has threatened our ability to treat common infectious diseases [1]. Moreover, antibiotic resistance is rising dangerously around the globe [2,3]. Several waterborne and foodborne diseases, such as pneumonia and tuberculosis-causing bacterial species [1], are becoming hard to treat due to the decrease in effectiveness of antibiotics [4]. Expeditious actions are required to halt the march towards a post-antibiotic era, which may lead to severe and commonly untreatable infections [4].

Several research studies have been undertaken to discover novel compounds that can eradicate the emergence of multi-drug-resistant bacteria through the improvement of antimicrobial properties that target specific cellular processes. However, these antimicrobial agents have drawbacks, such as the induction of adverse effects on human health, the minimum rate of response to treatment, and the possible development of resistance over time [5,6]. Therefore, there is a demand for new treatment strategies that can provide a pathway for developing antimicrobial therapeutics that cannot generate drug resistance in bacteria.

Plasma is a partially ionized gas, often known as the fourth state of matter [7]. Cold atmospheric plasma (CAP)-activated water has exhibited excellent antibacterial [8,9] and antiviral [10] properties [11]. Although there are plenty of studies that exhibit the anti-microbial aspects of plasma-activated water or other solutions, in this study we specifically used microwave plasma to generate reactive nitrogen species (RNS) in water and then observed its antimicrobial efficiency. Even though there are several research studies available [12] for plasma-treated water, there is still a need to understand the role of specific reactive oxygen and nitrogen species (RONS) dissolved in plasma-treated water, which play a significant role in sterilization or bacterial inactivation [11,13]. NO is one of the RNS generated by plasma that has been used for its antibacterial characteristics [11,14,15]. NO is a chemically inert, diatomic, radical gas molecule that can easily partition and diffuse through the cell and several other membranes in biological systems [16,17,18,19]. Owing to these properties, NO acts as a signalling molecule in eukaryotes and bacteria. In addition, NO reacts in complex ways with different molecules, metals, and other radicals to produce several other bioactive compounds that hinder enzymes, oxidize biomolecules, and arrest bacterial growth [8,14]. The inhibitory mechanism of NO comprises the collaborative action of several cellular regulatory processes, including amino-acid biosynthesis, central metabolism, oxidative phosphorylation, DNA replication, and DNA repair, ultimately resulting in a profound, nitrosative-stress-facilitated bactericidal effect [20]. Moreover, NO directly affects the structure of DNA, inhibits the DNA repair enzyme, and causes iron depletion and lipid peroxidation of polyunsaturated fatty acids (such as membrane phospholipids) in bacteria as it oxidizes and changes the conformations of cellular constituents and thus prevents normal functioning [21].

**Figure 1:**
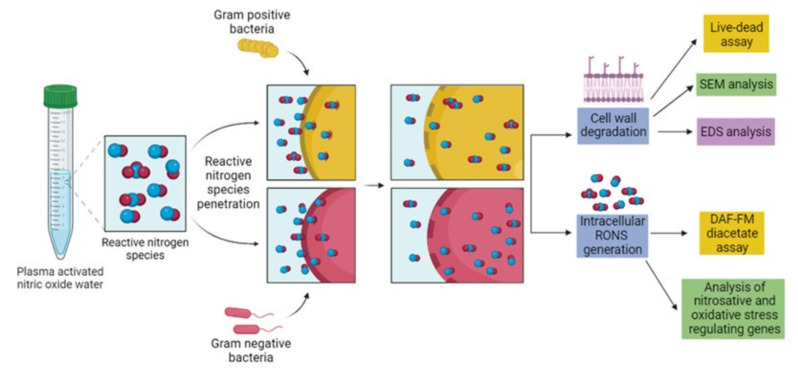
Schematic representation of antibacterial activity and mode of action of PG-NOW-generated RNS against pathogenic Gram-negative and Positive bacteria.

Microwave-generated, plasma-activated water produced at different parameters produces a cocktail of RONS and, according to these parameters, its antimicrobial potential also varies. Recently, it has been observed that PG-NOW has sanitizing properties as it inhibits bacterial skin infections [22]. In another study, microwave-generated, plasma-activated water showed a potential anti-viral action against the alpha-HCoV-229E [10] virus. Consequently, NO exhibits remarkable sterilization potential against several microorganisms including viruses, bacteria, fungi, and parasites [23]. However, it is essential to unveil the actual mechanism of the treatment of NO dissolved in PG-NOW on the bacterial system. The mechanism underlying the anti-bacterial effect of PG-NOW has not been explored yet, and PG-NOW can be used to overcome the development of resistance in bacteria [23]. The effect of PG-NOW on the oxidative and nitrosative stress-related gene expressions can provide an idea about the mode of action on cellular homeostasis mediated by PG-NOW-donated NO. In this study, we aim to understand the impact of NO-triggered stress on the bacterial cell and related stress response mechanisms by evaluating stress-response-related mRNA expression (Figure 1). Additionally, morphological changes were observed using SEM and an elemental analysis was conducted via energy-dispersive X-ray spectroscopy (EDS). Inclusively, we investigated the potentiality of PG-NOW-donated, NO-mediated nitrosative stress, subsequently leading to bacterial-growth inhibition.

## 2. Results

### 2.1. Physiochemical Characterization of PG-NOW

**Figure 2:**
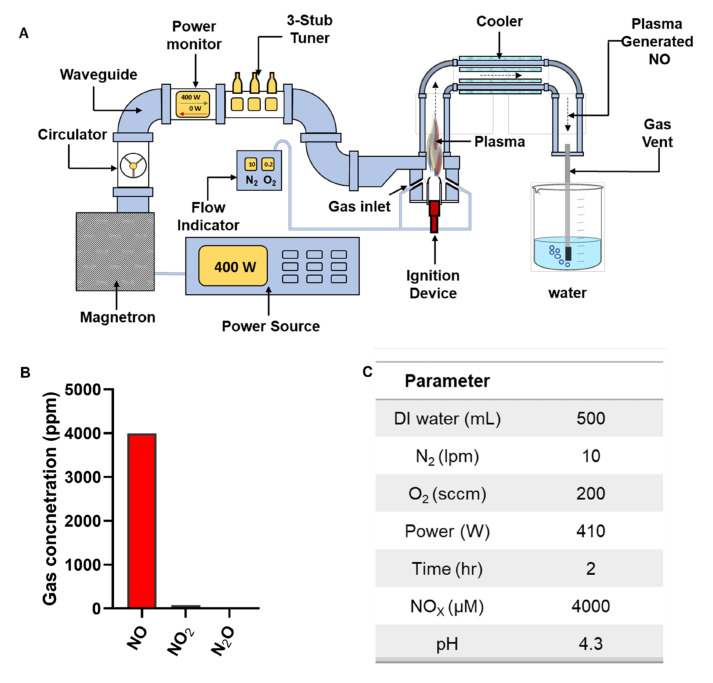
Treatment with PG-NOW. (**A**) Schematic representation of the generation of PG-NOW using gaseous NO by the microwave plasma system, (**B**) summary of the gaseous species’ composition concentration of the microwave plasma, as mentioned in our previous publication [10], and (**C**) parameters for generation of high NOx from gaseous NO exposed to DI water, as indicated and mentioned in our previous publication [10].

In this study, we used PG-NOW, which is synthesized using a microwave plasma system (shown in Figure 2A) using a mixture of oxygen- and nitrogen-swirl gas flowing at 200 sccm and 10 lpm (Figure 2C), respectively, as was previously described [10]. The microwave discharge of the microwave plasma system majorly generates a gaseous NO (Figure 2B), as was mentioned in our previous publication [10]. Other species, such as N_2_O and NO_2_, were also detected, but with much lower concentrations of 0.3 ppm and 70 ppm, respectively [10].

**Figure 3:**
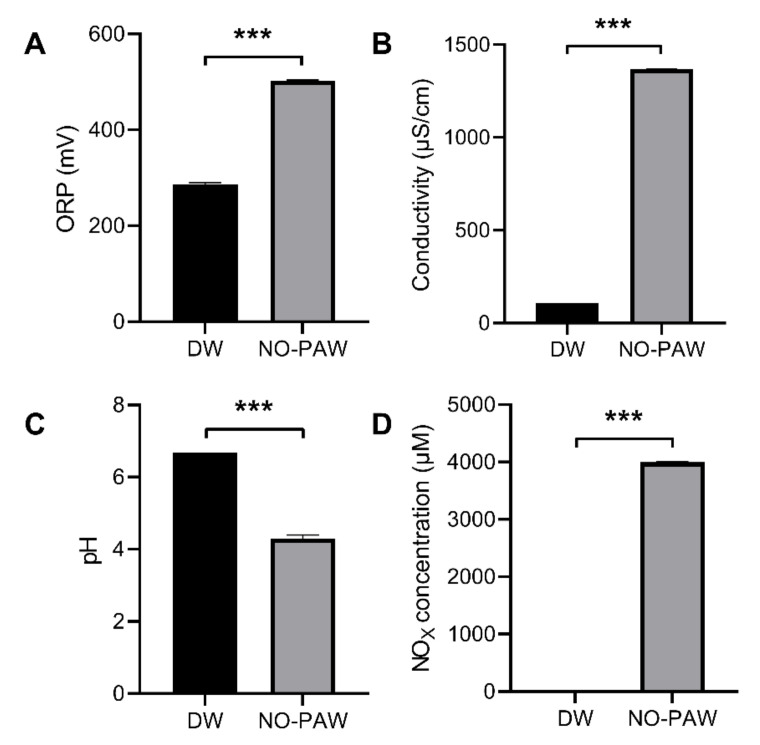
Physicochemical properties of PG-NOW generated using a microwave plasma system. (**A**) ORP, (**B**) conductivity, (**C**) pH, and (**D**) NO_X_ concentration of prepared PG-NOW using microwave plasma system, respectively. *** *p* < 0.001 vs. Control. The data are represented as the mean ± SD of three identical experiments with three replicates.

Based on the results of the gas-composition analysis using gas FTIR analysis, we speculate that NO with a concentration of at least 4000 ppm in the plasma afterglow can be injected into the liquid in the sealed system. We therefore used a commercialized NO-detection kit based on the Griess method to measure the NOx in the liquid phase, obtaining a concentration of 4000 uM. The interaction of plasma-generated NO with DI water altered the oxidation-reduction potential and pH, consequently increasing its conductivity [10,22]. As is shown in Figure 3, PG-NOW was generated using a microwave plasma system with an increased oxidation-reduction potential (ORP) of about 502 mV (Figure 3A) and an electrical conductivity (Figure 3B) of about 1367 µS/cm following treatment for 2 h with the microwave plasma system and a decreased pH of approximately 4.3 (Figure 3C). The amount of NO production always rises as the microwave power increases [10]. In our system, gaseous NO in the liquid phase slowly converted into other nitrogen oxides such as NO_2_^−^ and NO_3_^−^ [10]. The resulting NO species in the DI water signified that the total levels of NO, NO_2_^−^, and NO_3_^−^ are presented as NOx. The NOx produced was detected using a commercial QuantiChrom Nitric Oxide Assay Kit based on the improved Griess method. Furthermore, NO_X_ concentration was approximately 4000 µM in PG-NOW (Figure 3D), as was mentioned earlier [10].

### 2.2. Antibacterial Activity of PG-NOW

**Figure 4:**
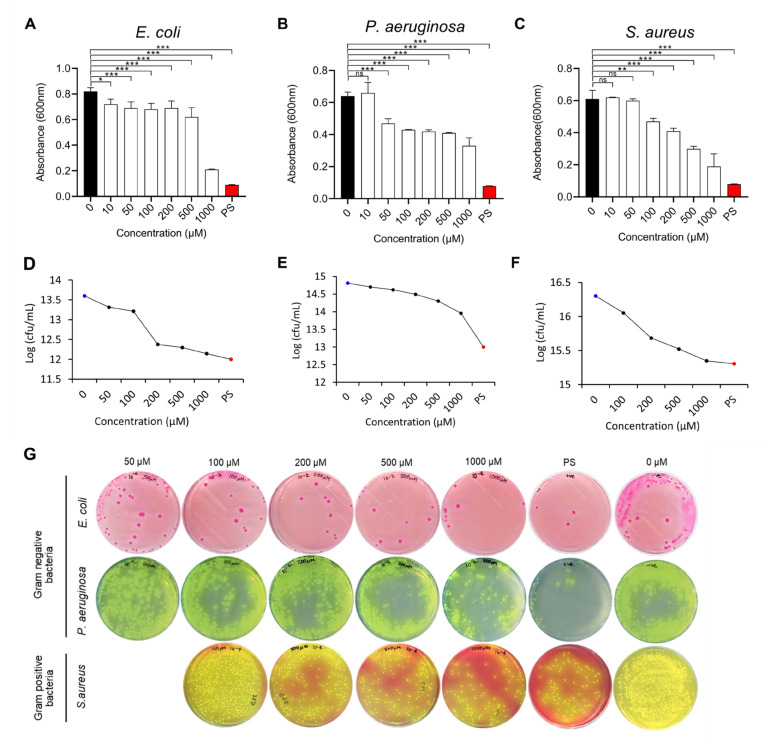
CFU assay of Gram-negative and Gram-positive bacteria treated with PG-NOW. (**A**–**C**) Determination of CFU using a microbroth dilution assay with a 1000, 500, 200, 100, 50, and 10 µM concentration of PG-NOW. Black bar displays the negative control and red bar displays the positive control; (**D**–**F**) Log CFU/mL; and (**G**) images of the CFU of three bacterial strains. * *p* < 0.05, ** *p* < 0.01, *** *p* < 0.001 vs. Control or untreated, ns = not significant. The data are represented as the mean ± SD of three identical experiments with three replicates.

In our study, we evaluated the bactericidal effects of PG-NOW enriched with NO against pathogenic bacterial strains of Gram-negative *E. coli* (K-12) [24] and *P. aeruginosa* [25] and Gram-positive *S. aureus* [26] (Table 1). The growth-inhibitory response of pathogenic bacteria was evaluated using a microdilution broth assay. The values plotted were based on the spectrophotometric assay and colony-forming units (CFUs). Treatment with the PG-NOW showed a dose-dependent effect on pathogenic-bacterial-growth inhibition (Figure 4A–C). For the CFU assay (Figure 4D–F), a log reduction in the CFU was observed following PG-NOW treatment of the pathogenic bacteria. The microdilution broth dilution assay demonstrated that *E. coli* (K-12) growth was particularly inhibited at the highest concentration of 1000 µM, followed by *S. aureus* and *P. aeruginosa*, with absorbances of 0.2, 0.2, and 0.3 at 600 nm wavelength, respectively. The CFU assay (Figure 4G), showed the 1.5, 0.9, and 1 log reductions in *E. coli* (K-12), *P. aeruginosa*, and *S. aureus* were equivalent to a 97, 86, and 90% percentage reduction in pathogen growth, respectively (calculated using https://www.omnicalculator.com/biology/log-reduction) (accessed on 1 September 2022). Our results demonstrated that there was a log reduction in Gram-negative and Gram-positive bacteria after treatment with PG-NOW.

**Figure 5:**
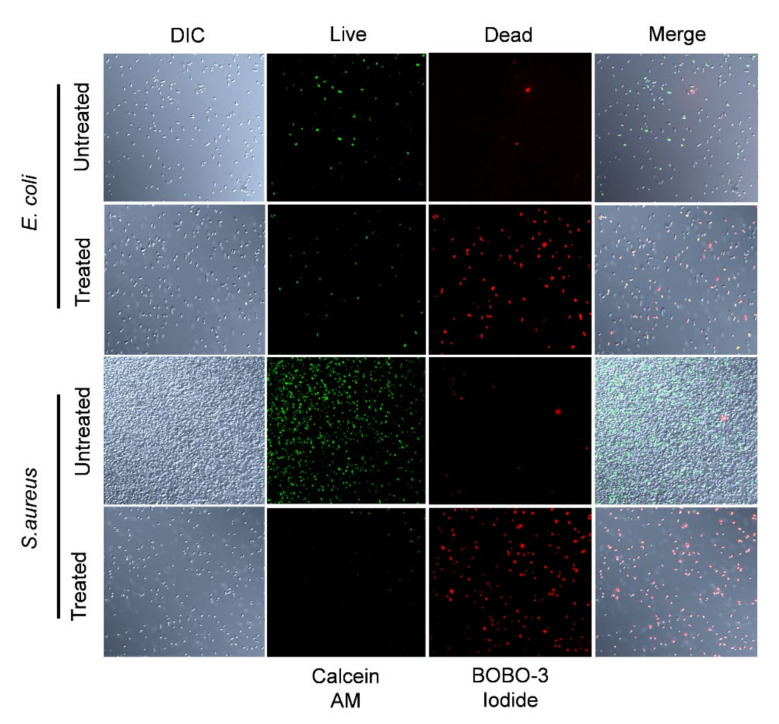
Bacterial cell integrity was compromised after PG-NOW treatment. Confocal laser scanning microscopy images show the live and dead cells of *E. coli* and *S. aureus* following PG-NOW treatment at 1000 µM concentration and stained with calcein AM (green fluorescence) and BOBO-3 iodide (red fluorescence), which indicates a compromised cell membrane. DIC: Differential Interference Contrast.

Previous studies showed that PG-NOW is effective against viruses [10] and bacteria [22]. Recently, research on plasma-activated water (PAWs) showed promising results, in which microbubble-enabled PAW at the lowest temperature had a higher antimicrobial activity against *E. coli* [27]. For the rapid quantitation of cell viability, a live and dead cell assay was performed. Live bacteria emitted green fluorescence and dead bacteria emitted red fluorescence; this was observed using confocal laser scanning microscopy. Reduction in the percentage of live cells of *E. coli *and *S. aureus* occurred in the 1000 µM PG-NOW-treated groups (Figure 5), indicating that the proportion of dead cells was increased due to loss of plasma membrane integrity, indicated using BOBO-3 iodide, which is a cell-impermeant dye that only binds to the DNA of bacteria with compromised cell membranes.

### 2.3. Analysis of Intracellular RNS Generated by PG-NOW in Bacteria

**Figure 6:**
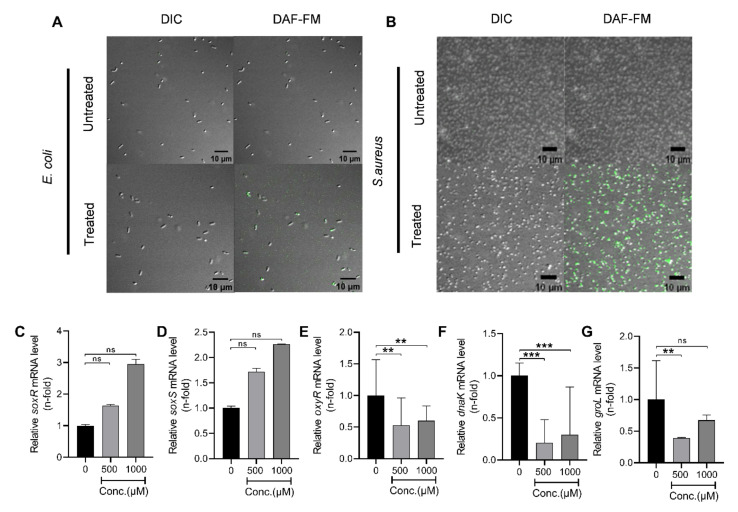
Reactive oxidative and nitrosative species formed by PG-NOW disrupt the homeostasis of bacterial cells. Intracellular levels of RNS in (**A**) *E. coli* and (**B**) *S. aureus* after PG-NOW treatment was observed using the DAF-FM DA assay, in which a fluorescent benzotriazole was seen. (**C**,**D**) Analysis of the nitrosative and oxidative stress regulons’ (*soxR* and *soxS)* mRNA expression in *E. coli*, and (**E**) *oxyR*, respectively. The heat shock genes (**F**) *dnaK*, and (**G**) *groL* mRNA expression following the PG-NOW treatment (1000 µM). ** *p* < 0.01, *** *p* < 0.001 vs. control or untreated, ns = not significant. The data are represented as the mean ± SD of three identical experiments with three replicates.

In this study, we investigated the RNS accumulation within bacterial cells post-PG-NOW-treatment. The reactive species generated by the PG-NOW can easily penetrate the cell membrane [28,29,30], subsequently hampering the bacteria’s homeostasis and leading to cell death. Data shown in Figure 6A,B exhibit the accumulated, intracellular RNS in *E. coli* and *S. aureus* following PG-NOW treatment. As is shown in Figure 6A,B, the untreated bacteria, *E. coli* and *S. aureus*, do not form a fluorescent benzotriazole and, consequently, demonstrate minimum fluorescence. Contrary to this, the PG-NOW-treated bacteria showed the presence of fluorescence, implying the generation of intracellular RNS.

### 2.4. Plasma-Activated NO Water Upregulates Nitrosative Stress Genes’ Response

To precisely detect the impact of PG-NOW treatment on bacterial cell homeostasis, damage repair, protein folding, oxidative, and nitrosative stress, we analyzed the mRNA expression of *dnaK*, *groL*, *oxyR* (oxidative stress response gene), *soxR*, and *soxS* (superoxide response regulon) genes, respectively [31], in *E. coli* (shown in Figure 6C–G) [32,33,34,35]. The data shown in Figure 6C,D exhibit the mRNA expression of the *soxRS* gene. The mRNA expression of *soxR* and *soxS* in the post-PG-NOW treatment group (500, 1000 µM) showed a dose-dependent increase in contrast to the control group, which implies the activation of *soxRS* genes induced by rising superoxide levels. Moreover, the higher the concentration of the PG-NOW treatment, the higher the upregulation of the *soxR* and *soxS* genes was observed. This indicates the regulation of nitrosative stress in the cell [36]. Similarly, oxidative stress can damage the nucleic acids and proteins, and can impede the protein functions [37]. To analyze the intracellular oxidative stress, we evaluated the mRNA expression of the oxidative-stress-related gene *oxyR* [38]. The *oxyR* gene expression was less when compared to the control group. It was not elevated, clearly implying the negligible production of H_2_O_2_ stress (Figure 6E). Evidently, the generation of oxidative stress in bacterial cells was less because the PG-NOW had a negligible amount of H_2_O_2_, as was previously observed [10].

In addition, we analyzed the *dnaK* mRNA expression of a stress-induced cellular chaperone, the DnaK chaperone, which, along with its co-factors, is essential in de novo protein folding [34]. In our study, the mRNA expression of *dnaK* (Figure 6F) and *groL* (Figure 6G) was down-regulated after PG-NOW treatment (500, 1000 µM) when compared to the control treatment group, demonstrating that the PG-NOW treatment hampered the *E. coli* bacteria chaperone system, which is prerequisite for bacterial survival during normal physiological and stress-induced conditions.

### 2.5. SEM and EDS Analysis

**Figure 7:**
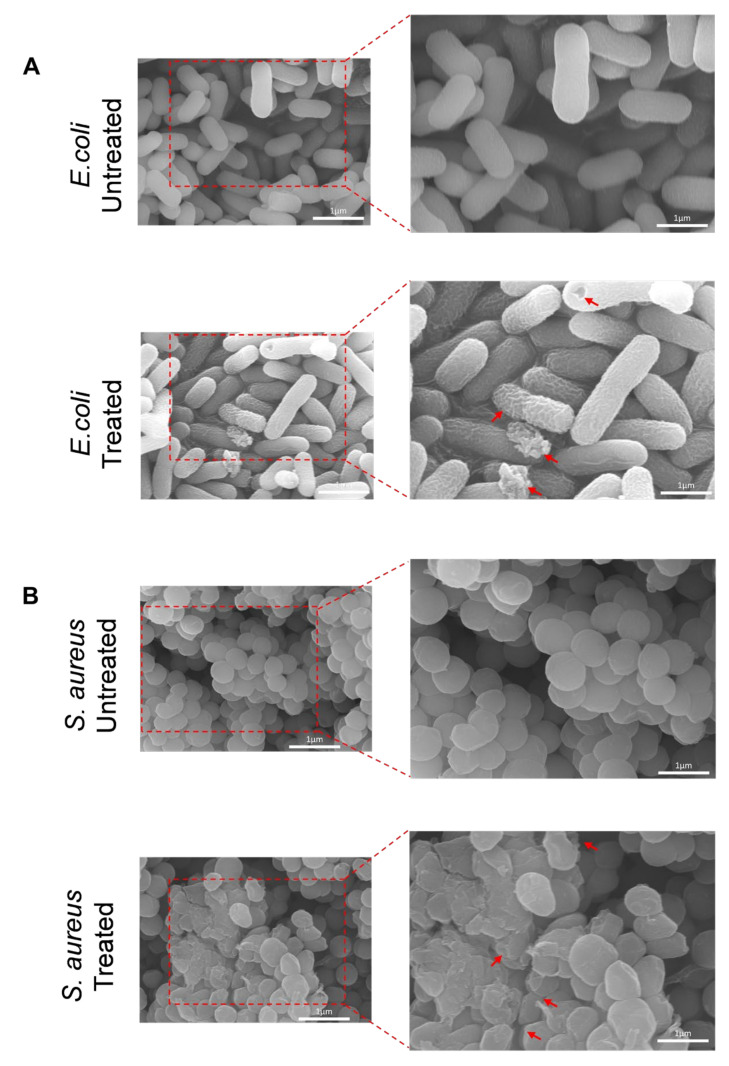
SEM images of PG-NOW untreated and 1000 µM-treated (**A**) *E. coli* (K-12) and (**B**) *S. aureus*, showing deformed structures, pores, and appearing corrugated (indicated with red arrows).

To further understand the inactivation mechanisms of PG-NOW, SEM images of bacteria were examined. The untreated and treated surface morphologies of PG-NOW treated bacteria samples is illustrated in Figure 7. The bacterial surface was analyzed at different magnifications of a 1 µm diameter: 15,000× and 20,000×, shown in Figure 7. The observation of the SEM images showed that the untreated *E. coli* and *S. aureus* samples appeared as cylindrical, smooth, and with a circular, smooth surface. When they were treated with PG-NOW, the cell surface was deformed, appeared corrugated, and showed pores in the cell wall. However, the bacterial cell shrinkage and debris formation was specifically caused by the effect of the PG-NOW-generated RNS.

**Figure 8:**
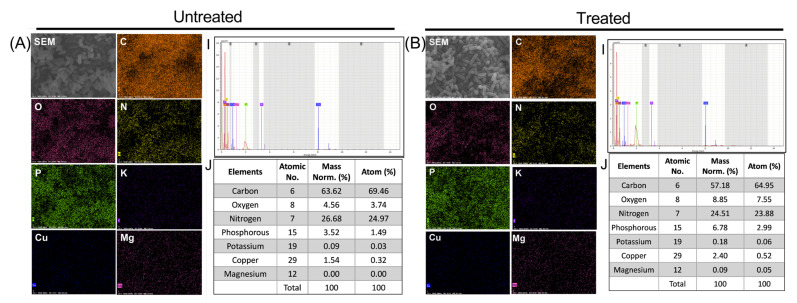
EDS elemental mapping displayed the distribution of elements in the PG-NOW untreated (**A**) and treated samples (**B**) of the *E. coli* strain, which shows the SEM image of the bacterial structure together with carbon (C), oxygen (O), nitrogen (N), phosphorous (P), potassium (K), copper (Cu), and magnesium (Mg)and their spectra with mass percentage.

**Figure 9:**
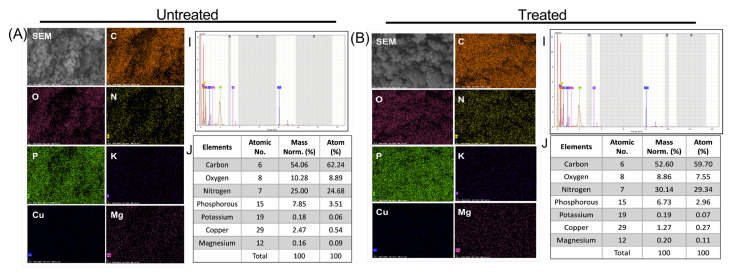
EDS elemental mapping displayed the distribution of elements in the PG-NOW untreated (**A**) and treated samples (**B**) of the *S. aureus* strain, which shows the SEM image of the bacterial structure together with carbon (C), oxygen (O), nitrogen (N), phosphorous (P), potassium (K), copper (Cu), and magnesium (Mg) and their spectra with mass percentage.

In addition, for a better understanding of the damage caused by the PG-NOW on the bacteria, we performed an elemental analysis. Therefore, EDS was used to confirm the effect of PG-NOW on the chemical composition of a bacterial cell, which was examined in the untreated and treated samples, as is shown in Figure 8 and Figure 9. EDS analysis was used to detect the variations in elements after the interaction of NOx species with the bacteria cells post-PG-NOW treatment in comparison to the untreated control (Figure 8 and Figure 9). Using an EDS protocol, the quantitative analysis of the EDS-collected images of *E. coli* identified the presence of a collective, normalized mass that consisted of several elements. A significant difference was observed in the case of carbon (63.62%), oxygen (4.56%), nitrogen (26.68%), and phosphorus (3.52%), which showed changes after PG-NOW. These changes include composition changes, such as for carbon (57.18%), oxygen (8.85%), nitrogen (24.51%), and phosphorus (6.78%), as is shown in Figure 8. Similarly, in *S. aureus*, the collective, normalized mass in untreated samples was carbon (54.06%), oxygen (10.28%), nitrogen (25.00%), and phosphorus (7.85%), which was changed after treatment to values of carbon (52.60%), oxygen (8.86%), nitrogen (30.14%), and phosphorus (6.73%), as is shown in Figure 9.

## 3. Discussion

Bacteremia and bacterial infections have risen globally due to antimicrobial resistance. In addition, these common bacterial pathogens—namely *E. coli*, *P. aeruginosa*, and *S. aureus*—attribute to the rise in bacteremia and related diseases. *E. coli* and *P. aeruginosa* belong to a Gram-negative family. *E. coli* is often present in water and food, in addition to being used as an indicator of fecal contamination due to the fecal-material deposition in the environment by warm-blooded animal intestines, which impacts the environment [39]. *P. aeruginosa* is present in water and soil. It is also an indicator of fecal contamination and is easily found in the environment. It causes opportunistic infections that cause biofilms [40]. Similarly, *S. aureus* is Gram- negative bacteria that survives in the environment on inanimate surfaces for prolonged periods and is present in water, air, and food. It is an opportunistic pathogen that colonizes on skin and may cause life-threatening infections [41]. Therefore, there is an urgent need for the eradication or control of these pathogenic bacteria in the environment. In this study, we used microwave CAP for the preparation of NO-enriched plasma water for the eradication of these bacterial pathogens.

CAP has been an extensively used for several applications, such as in agriculture, medicine, etc., with substantial evidence for its efficient bactericidal action against various pathogens [22,42,43,44]. Recently, plasma-treated liquids have shown promising effects in plasma medicine and agriculture, similar to the effects of direct CAP treatment [7,9,10,11,22,45,46]. The ability of plasma-activated water to restrict bacterial growth is gained via the formation of RONS following plasma discharge in water, in which the RONS increase the redox potential and conductivity and decrease the pH, creating an acidic environment that favors effective bacterial inhibition [13,27]. As was previously mentioned, the PG-NOW generated by our microwave plasma system produced a high amount of gaseous NO at 1700–2000 cm^−1^. In contrast, NO_2_, and N_2_O concentrations were significantly less: about 75 ppm and 0.3ppm, respectively [10]. The freshly prepared PG-NOW showed a concentration of NO of approximately 4000 µM (Figure 2B), which is entirely reliant on the parameters (Figure 2C) set during the generation of plasma. Therefore, studies on plasma-treated liquids have revealed promising results and assessed plasma-treated liquids as substitutes for existing antibacterial agents in agriculture, food, and medicine.

NO is chemically active and can interact with various biological molecules, easily penetrating the lipid bilayer, the hydrophobic region of proteins’ cell membranes; it is for this reason that NO has been studied for its bacteriostatic activity [14]. The reactive byproducts of NO exhibit antibacterial action via several mechanisms such as lipid peroxidation, protein modification, and DNA degradation that leads to cell death [47,48,49,50]. Particularly, the ONOO- and peroxynitrous acid (ONOOH) can permeate easily through phospholipid membranes, whereas ONOO- reacts with biological substances such that the reaction results in the nitration of tyrosine in proteins [51] and the oxidation of methionine [52], lipids [53], DNA [54], and redox metal centers [55,56]. Due to the high hydrophobicity of NO, it is ten times more readily soluble in hexane than in water [30]. Its high diffusion rate in lipophilic milieu [57] and lipophilic [58] behavior reveal its crucial role in lipid oxidation and membrane modification [59]. The exposure of exogenous NO and its byproducts to pathogens proved to be beneficial for the their growth inhibition properties without generating resistance [60]. For instance, repeated exposure to NO did not stimulate resistance in *P. aeruginosa* and *Methicillin resistant Staphylococcus aureus* [60]. In this regard, NO alone can exhibit bactericidal action without any adverse resistance. Additionally, to the best of our knowledge, plasma-generated NO can have advantages compared to bottled NO, which can be a highly toxic gas that may cause serious damage to the human body upon inhalation. Thus, a pure NO tank (market available) is not suitable for biomedical applications. In practice, NO is stored in a gas tank at a low concentration (up to a few thousand ppm) with Nitrogen as a balance gas. It is also expensive due to its production, storage, and transportation costs. On the other hand, our plasma system can effectively generate high concentrations of NO from N_2_ and O_2_. This is a potential approach for production and broadens the range of applications of plasma-generated NO.

Recent research studies have demonstrated that a microwave-plasma-generated liquid exhibits excellent antibacterial activity due to the generation of high-density electrons and active species [22]. In our study, we explored the antibacterial potential of PG-NOW generated using our microwave plasma system. Our results demonstrate that the PG-NOW treatment on both Gram-positive and Gram-negative bacteria has shown significant CFU-log reductions. We evaluated the bactericidal effects of PG-NOW against gram-negative *E. coli* (K-12) and *P. aeruginosa* and gram-positive *S. aureus. E. coli* and *S. aureus* showed a significant log reduction in the CFU of 4.456 and 3.951, respectively, which was equivalent to a 99.9% reduction in growth. Previously, microwave-generated plasma water has been investigated for its antibacterial potential, such as a disinfectant [22] and for the inactivation of biofilms [61].

The ability of plasma water to inhibit pathogens is gained by adjusting the various parameters that generate reactive species. Different parameters were tested for the generation of PAW to unveil the mechanism of inactivation processes, after which it was found that the combination of a low pH, high ORP, and various reactive species are responsible for the antibacterial activity [62], which is similar to the results of our study. Similar to Qian-Yun Han et al. [62], our PG-NOW has a low pH, high ORP, and consists of various RNS that resulted in a bactericidal effect against Gram-negative and Gram-positive bacteria. NO is the major component that plays a significant role in the bactericidal action in our study. The capacity of exogenous NO species to disrupt the bacterial cell membrane has already been studied, in which NO species were incorporated in chemically modified chitosan oligosaccharides that exerted a broad-spectrum antibacterial activity [63]. Further, we analyze the percentage of live and dead bacteria using a live and dead assay, in which 1000 µM of PG-NOW showed bacteria with compromised cell membrane.

Oxidative stress is caused by an imbalance between the endogenous reactive oxygen species (ROS) and the biological system’s ability to detoxify them and their intermediates [25]. To mitigate the damage of nitrosative and oxidative stress on bacteria, several stress-response regulon genes are activated. The interaction of RNS with the bacterial membrane and further intervention with the bacterial homeostasis has resulted in bacterial death. To investigate this phenomenon, we investigated the intracellular RNS generation post-PG-NOW treatment using the DAF-FM DA assay. In addition, we analyzed the genes related to homeostasis and nitrosative and oxidative stress. DAF-FM DA is a cell-penetrable deacetylated form of DAF-FM that is hydrolyzed by bacterial intracellular esterases into cell-impermeable DAF-FM after a reaction with an RNS. In this assay, untreated *E. coli* and *S. aureus* showed less or negligible green fluorescence, indicating decreased intracellular RNS generation. On the contrary, treated bacteria showed a high green fluorescence, indicating the generation of intracellular RNS.

Moreover, we analyzed the nitrosative-, oxidative-, and homeostasis-stress-related genes *soxR*, *soxS*, *oxyR*, *dnaK*, and *groL* in *E. coli* mRNA expression. The SoxR protein is merely activated by increased superoxide levels that subsequently activates the *soxS* gene transcription, leading to the synthesis of the SoxS protein, consequently resulting in the activation of approximately ten genes [64]. In our study, *soxRS* gene expression was dose-dependent and elevated post-PG-NOW treatment (500 and 1000 µM). This implies the intracellular nitrosative stress generation in the bacteria that lead to cell death [65,66].

OxyR, is a transcription factor that is widely seen in Gram-negative bacteria. It is directly induced by H_2_O_2_. The activation of OxyR proteins recruits RNA polymerase to transcribe approximately thirty stress-response genes [67]. Moreover, OxyR is responsible for regulating detoxification for protein-damage repair [67]. The data shown in Figure 6E show a decrease in the *oxyR* expression, which implies that *oxyR* is activated by concentrations of H_2_O_2_ beyond 0.1µM [68]. In our study, the concentration of H_2_O_2_ in the PG-NOW was possibly not sufficient to elicit and activate the *oxyR* expression in *E. coli.*

The two major chaperone systems in *E. coli* are GroELS and DnaK [33,69,70,71,72]. DnaK binds to its cofactor DnaJ, which is an obligatory reaction that accelerates the hydrolysis of ATP attached to DnaK, which is essential for binding DnaJ to the polypeptide chain and to protect polypeptide chain from misfolding. GroEL is a double-ring-shaped oligomeric 14-mer chaperonin that binds and encapsulates naïve protein chains. Along with its co-chaperonin GroES, it caps the central cavity of GroEL and facilitates the correct folding of naïve proteins in the presence of ATP [71]. In this study, the *dnaK* and *groL* gene expressions were down-regulated when compared to control group, implying that, after PG-NOW treatment, the cell homeostasis collapsed due to chaperone malfunctioning and high intracellular-RNS generation leading to cell death. It is found that ONOO- impairs the GROEL chaperone activity via protein modifications; similarly, it might have halted the *dnaK* [73] and *groL* [74] gene expressions in our study, as was observed after PG-NOW treatment.

To further investigate the bacterial deactivation, we evaluated the microscopy-based SEM and elemental analysis. The microscopy-based SEM analysis facilitated the study of the effect of antibacterial treatment on cell structure. Bacterial-morphological changes were observed from SEM images post-PG-NOW treatment, similar to several studies conducted earlier [75,76]. Xiang, Q. et al. [75] compared the SEM images of bacteria before PAW treatment and post-PAW treatment: the bacteria initially had an intact cell structure and were rod-shaped with smooth surfaces. However, after treatment with PAW for 10 min, the bacterial cells showed distinguishable changes in their morphology. Similar to our study, transitions from smooth surfaces to distortion, corrugation, shrinkage, and ruptured surfaces were observed (Figure 7).

In the elemental analysis of untreated and treated *E. coli* and *S. aureus*, we observed changes in the collective elemental mass after the PG-NOW treatment. We evaluated several elements, but a significant difference was observed in the cases of carbon, oxygen, and nitrogen after treatment. In *E. coli*, the masses included carbon: (66.66%) and oxygen (4.78%), which were elevated to carbon (68.40%) and oxygen (7.70%). Nitrogen was slightly decreased, going from 27.95 to 22.61%. Similarly, in *S. aureus*, the collective elemental mass was carbon (21.99%), oxygen (4.18%), and nitrogen (10.17%), and this increased to carbon (57.72%), oxygen (9.72%), and nitrogen (33.08%) after treatment. This elevation indicated that the RNS generated in PG-NOW might have interacted with the bacteria cell membrane, leading to a rise in the above elements. The microwave plasma torch temperature was about 6000 K. Under this condition, molecular nitrogen (N_2_) and oxygen (O_2_) dissociate into atomic N and O, respectively. NO is then formed through a thermal mechanism (Zeldovich mechanism) through the following reactions [77]:

N_2_ + O → NO + N (1)

N + O_2_ → NO + O (2)

The overall reaction can be rearranging as:

N_2_ + O_2_ → 2NO (3)

In conclusion, our study determined the excellent antibacterial effect and the mechanism of action of PG-NOW against environmentally transmitted pathogens. It is essential to further investigate and explore the effect of PG-NOW on other bacterial pathogens. PG-NOW can be classified as a novel antimicrobial agent without the generation of resistance.

## 4. Materials and Methods

### 4.1. Microwave Plasma System and PG-NOW Preparation

Figure 1A demonstrates the microwave plasma system which generates the NO and PG-NOW, whose configuration was described earlier [11]. The microwave plasma system is comprised of a magnetron (i.e., microwave generator), waveguide parts, and a microwave plasma torch comprised of a quartz tube. The microwave plasma was generated by applying nitrogen (10 lpm) and oxygen (200 sccm) as a swirling gas. The swirling gas was injected into the system and adjusted using a mass-flow controller. In this experiment, the microwave power was operated at approximately 400 W, which generated the plasma torch in the quartz discharge tube. The NO gases generated in the plasma torch flame, including NO, NO_2,_ and N_2_O, entered a cooler, which lowers the temperature of the gas. The cooled gases were introduced into the deionized (DI) water. The microwave discharge of the microwave plasma system generated a cocktail of NO species in the liquid phase through the dilution of NO into DI water, forming various nitrogen oxides such as NO_2_^−^ and NO_3_^−^ [10] (Figure 1B–C). The physiological characteristics of the untreated DI water was assessed: the electrical conductivity was observed at 108 µS/cm, with a pH of approximately 6.6 and an ORP of approximately 286 mV. The characterization of PG-NOW was carried out, as was mentioned earlier [10].

### 4.2. Characterization of PG-NOW

The total NO_X_ (NO, NO_2_, and NO_3_) concentration in PG-NOW was determined by using a commercial QuantiChrom NO Assay Kit (QuantiChrom Nitric Oxide Assay Kit, BioAssay Systems, Hayward, CA, USA) based on the enhanced Griess method. All measurements were performed following the instruction of the manufacturers. The ORP and conductivity of the samples were analyzed by an ORP30 and CON30 Tester (Clean Instruments, Shanghai, China), respectively. The pH value of the PG-NOW samples was obtained using a pH spear (Eutech Instruments, Paisley, United Kingdom).

### 4.3. Determination of Cell Inhibition with Colony-Forming Unit Assay

*Escherichia coli* K-12 (*E. coli*) (KCTC 1116), *Pseudomonas aeruginosa* (*P. aeruginosa*) (KCTC 1636), and *Staphylococcus aureus* (*S. aureus*) (ATCC 12600) were used for the investigation of the antibacterial assay (Table 1). The colony-forming unit assay was used for the determination of growth inhibition of the bacterial CFUs [78]. Briefly, a log-phase culture of the bacterial strains was diluted to obtain a final concentration of 1 × 10^5^ CFU/mL [78] in tryptic soy broth (TSB) (MB-T1053, MB cell, Gyeonggi-do, Republic of Korea). The PG-NOW was diluted to obtain a range of different concentrations of NOx (1000, 500, 200, 100, 50, and 10 µM) in microplates. Medium containing only bacteria was used as negative control, and penicillin-streptomycin (PS) (LS203-01; Welgene, Gyeongsan-si, ROK, Republic of Korea) was used as the positive control. The microplates were incubated at 37 °C overnight, and their absorbance at 600 nm was measured using a microplate reader. The treated and untreated bacterial suspensions were spread on selective agar, such as MacConkey agar (MB-M1028; MB-cell, Gyeonggi-do, Republic of Korea) (for *E. coli*), cetrimide agar (MB-C2137; MB-cell, Gyeonggi-do, Republic of Korea) for *P. aeruginosa*, and mannitol salt agar (MB-M1029; MB-cell, Gyeonggi-do, Republic of Korea) for *S. aureus*. They were then incubated overnight at 37 °C, and the CFU was calculated as follows:

$$\frac{\mathrm{Number}\;\mathrm{of}\;\mathrm{colonies}\;\mathrm{on}\;\mathrm{plate}\;\times \;\mathrm{dilution}\;\mathrm{factor}\;}{\mathrm{The}\;\mathrm{volume}\;\mathrm{of}\;\mathrm{culture}\;\mathrm{plated}\;\mathrm{in}\;\mathrm{mL}}=\;\mathrm{Number}\;\mathrm{of}\;\mathrm{bacteria}/\mathrm{mL}$$

The CFU is the calculation of viable bacterial cells. Serial dilutions were used to calculate the concentration of bacteria in the original sample. As it would usually be impossible to count the number of bacteria in an original sample (with high number of bacteria), the bacterial sample was diluted and plated to obtain a reasonable number of colonies to count. Each dilution was plated on an agar plate. The number of bacteria per ml in the original bacterial stock was then calculated by multiplying the dilution factor.

### 4.4. Live–Dead Assay

To distinguish the live and dead cells, PG-NOW-treated and untreated bacteria were stained with a LIVE/DEAD™ Cell Imaging Kit (R37601) (Invitrogen™, Thermo Fisher Scientific, Waltham, MA, USA) according to the manufacturer’s protocol. *E. coli* and *S. aureus* were grown in TSB media and treated with a final concentration of 1000 µM of PG-NOW. The treated and untreated cells were pelleted at 10,000 g × 10 min and washed three times with a 0.85% sterile sodium chloride (NaCl) (S9888, Sigma-Aldrich, St. Louis, MO, USA) solution. Finally, the pelleted bacteria were resuspended in an equal volume of saline. A 2× working solution of live green and dead red was added, and the bacteria were further incubated for 15 min at a temperature between 20 and 25 °C in the dark. The cells were imaged using confocal laser scanning microscopy. The non-fluorescent calcein AM is a cell-permeable dye which is enzymatically converted to fluorescent calcein AM, emitting green fluorescence based on its esterase activity. The BOBO-3 iodide is a cell-impermeable dye that enters compromised membranes, producing a bright red fluorescence upon binding to DNA.

### 4.5. Detection of Intracellular RNS

Herein, the detection of intracellular RNS was carried out by DAF-FM DA (D23844, Invitrogen™, Thermo Fisher Scientific, Waltham, MA, USA) according to the manufacturer’s protocol. DAF-FM DA is a cell-permeable, deacetylated form of DAF-FM. After a reaction with an RNS, it is hydrolyzed by intracellular esterases into cell-impermeable DAF-FM. An imbalance of intracellular RNS causes nitrosative stress in the bacterial cell [79]. NO has a significant role in the dispersion of the biofilm in bacteria [15]. However, the intense oxidation–reduction reaction of the RNS damages the varieties of signaling biomolecules. In addition, an excess of them can jeopardize their homeostasis. Consequently, bacterial cells possess defense mechanisms to manage nitrosative stresses [31,37,64,65,66,67]. In addition, we also analyzed the mRNA expression of nitrosative stress regulon genes *soxR* and *soxS*, which are triggered by NO stress in untreated and treated cells.

### 4.6. Quantitative Real-Time PCR Analysis

In this study, RNA from bacterial cells was extracted using a TRI reagent (T9424) (Sigma-Aldrich, St. Louis, MO, USA) following the protocol provided by the manufacturer. RNA quantification was performed, and total 2 µg of RNA was used to synthesize cDNA by using a MMLV Reverse Transcriptase supermix kit (RT001M, Enzynomics, Daejeon, Republic of Korea) and a thermocycler (Applied Biosystems, USA), following manufacturer’s guidelines. Quantitative qPCR was performed using iQ SYBR Green Supermix (170-8882; Bio-Rad, Hercules, California, USA) on a real m-time PCR-detection system (Bio-Rad, Hercules, CA, USA). All the primers listed in Table 2 were purchased from DNA Macrogen (Seoul, Republic of Korea).

### 4.7. SEM and EDS

To obtain morphological data, the bacterial cells were analyzed using SEM following the PG-NOW treatment. The cells were pelleted and washed 3× with phosphate buffer saline (PBS) (LB001-02, Welgene, Daegu, Republic of Korea), undergoing 3500 rpm for 10 min at 4 °C. Further, cells were fixed with 4% (*v*/*v*) paraformaldehyde (Biosesang, Seongnam, Republic of Korea), overnight at 4 °C. Cells were again washed 3× with PBS, undergoing 3500 rpm for 10 min at 4 °C. They were re-fixed in 1% Osmium tetroxide (OsO_4_) (18459, Ted Pella Inc., Redding, CA, USA) in PBS for 48–72 h (h) at room temperature. Subsequently, cells were dehydrated by resuspending them sequentially with a range of ethanol concentrations %*v*/*v* (30%, 50%, 70%, 80%, and 100% *v*/*v* Ethanol) for 10 min each rinse, along with a final dehydration step of incubation in 100% ethanol for 15 min. For chemical drying, the samples were maintained in hexamethyldisilane (HMDS) (EMS16700, Madrid, Spain) for 5 min at room temperature. The samples were then dried in a desiccator overnight and sputter-coated with gold (108 Auto/SE Sputter Coater, Ted Pella Inc., USA). The samples were viewed under a SEM (JEOL, JSM-7001F, Tokyo, Japan) at an accelerating voltage of 15 kV. In addition, EDS (XFlash 6I60, Bruker, Billerica, MA, USA) was carried out for elemental compositional analysis of PG-NOW-treated *E. coli* and *S. aureus*. To obtain the elemental composition with a quantitative percentage of the untreated and treated bacteria, we performed an EDS analysis (Bruker, Germany). We analyzed the elemental compositions using an SEM monitor integrated with EDS.

### 4.8. Statistical Analysis

The recovered data are displayed as the mean ± SD. Statistical analysis was performed via student *t*-tests for ORP, conductivity, pH, and NOX and by one-way ANOVA for *soxRS*, *oxyR*, *groL*, and *dnaK*. Results were considered significant when * *p* < 0.05, ** *p* < 0.01, and *** *p* < 0.001. ns = not significant.

## 5. Conclusions

In this study, we have examined the role of PG-NOW prepared using a microwave plasma system in bacterial inhibition. PG-NOW has been previously reported to inhibit 229E coronavirus [10]. Our findings indicated that PG-NOW prepared using gaseous NO shows promising results for the inhibition of pathogenic bacterial growth. PG-NOW-generated RNS interacted with the bacterial membrane, resulting in corrugated membrane and cell structure disintegration. This was observed via the mRNA expression of oxidative and nitrosative stress genes and an SEM analysis. The outcomes of the present study could instigate the motivation to develop a PG-NOW-based antibacterial treatment system without causing resistance in bacteria. This comparatively affordable strategy could be used in a clinical setting. PG-NOW could be administrated through oral, intratracheal, or intraperitoneal delivery or in a combination for the treatment of bacterial infection. Future clinical study regarding this topic is required to further improve our understanding of the effective and safe dosage levels and the biocompatibility of PG-NOW for antibacterial therapeutics. For this reason, future research work should focus on discovering the pharmacokinetic parameters of PG-NOW treatment technologies for their safe administration.

## Figures and Tables

**Table 1:** Bacteria used in the study and their pathogenic properties.

Strain	Gram Nature	Pathogenic Properties	Origin
*E. coli* (K-12)	Negative	Cause illness, diarrhoea, dysentery, meningitis, urinary tract infection, etc.	Waterborne; foodborne
*P. aeruginosa*	Negative	Cause nosocomial infections such as pneumonia, urinary tract infections, surgical wound infection, bacteraemia, etc.	Waterborne; soilborne
*S. aureus*	Positive	Develops multiple antibiotic resistance, bacteraemia, infective endocarditis, osteoarticular, skin and soft tissue, pleuropulmonary, etc.	Waterborne; Airborne; foodborne

**Table 2:** Primer list used in the study.

Gene Name	Sequence (5′-3′)	Size (bp)
*gapA*-forward	CACGCTACTACCGCTACTCA	205
*gapA* -reverse	CGGTCAGGTCAACTACGGAT	
*sorR*-forward	GTATCGGCGCTGCATTTCTA	208
*soxR*-reverse	GCTGTTTCCACTCTTTCGCA	
*soxS*-forward	GCATATTGACCAGCCGCTTA	245
*soxS*-reverse	GATCAAACTGCCGACGGAAA	
*oxyR*-forward	AGTTGGACCGTACCTGCTAC	161
*oxyR*-reverse	CTTCGCTCTCTTTCACCAGC	
*groL*-forward	TCCGTACCATGCTCTGACTC	178
*groL*-reverse	GCATACCTTCAACCACGTCC	
*dnaK*-forward	ATCGAACTGTCTTCCGCTCA	156
*dnaK*-reverse	AACTTTCAGCGGCTCAATGG	

## Data Availability

The data presented in this study are available on request from the corresponding author.
